# A Semi-Pilot Photocatalytic Rotating Reactor (RFR) with Supported TiO_2_/Ag Catalysts for Water Treatment

**DOI:** 10.3390/molecules23010224

**Published:** 2018-01-20

**Authors:** Carlos Montalvo-Romero, Claudia Aguilar-Ucán, Roberto Alcocer-Dela hoz, Miguel Ramirez-Elias, Victor Cordova-Quiroz

**Affiliations:** Department of Chemical Sciences, Universidad Autónoma del Carmen, Calle 56 No. 4, Avenida Concordia, Ciudad del Carmen, Campeche 24180, Mexico; cmontalvo@pampano.unacar.mx (C.M.-R.); roberto.alcocer.hoz@gmail.com (R.A.-D.h.); mramirez.unacar@gmail.com (M.R.-E.); acordova@delfin.unacar.mx (V.C.-Q.)

**Keywords:** rotating photocatalytic reactor, TiO_2_/Ag catalysts, water treatment

## Abstract

A four stage semi-pilot scale RFR reactor with ceramic disks as support for TiO_2_ modified with silver particles was developed for the removal of organic pollutants. The design presented in this article is an adaptation of the rotating biological reactors (RBR) and its coupling with the modified catalyst provides additional advantages to designs where a catalyst in suspension is used. The optimal parameter of rotation was 54 rpm and the submerged surface of the disks offer a total contact area of 387 M^2^. The modified solid showed a decrease in the value of its bandgap compared to commercial titanium. The system has a semi-automatic operation with a maximum reaction time of 50 h. Photo-activity tests show high conversion rates at low concentrations. The results conform to the Langmuir heterogeneous catalysis model.

## 1. Introduction

Advanced oxidation processes (AOP) are widely used for water decontamination. Such processes rely on the photocatalysis principle, that is, the generation and use of the hydroxyl radical (·OH) in the presence of UV light with an adequate intensity. The photocatalytic process can reduce the initial organic matter to CO_2_, H_2_O and mineral salts. This technology has proved its efficiency for the complete elimination of different organic molecules.

The mechanism of the process of oxidation/reduction using TiO_2_ can be described through the following reactions [1,2,3] (Equations (1)–(7)):

(1) $$TiO_{2}\overset{hv}{\to }e^{-}+h^{+},$$

(2) $$e^{-}+h^{+}\to Heat,$$

(3) $$h^{+}+H_{2}O\to HO•+H^{+},$$

(4) $$h^{+}+HO^{-}\to HO•,$$

(5) $$h^{+}+D_{ads}\to D^{+},$$

(6) $$e^{-}+A_{ads}\to A^{-},$$

(7) $$HO•+D\to D_{\;oxidized}.$$

Some reactions generated during a catalyst activation process with UV light can interfere directly in the overall efficiency; as the reaction of charge recombination occurs when the removed electron (e^−^) from a photo-generated hole (h^+^) is combined between generating neutral centers (Reaction (2)), this process can be reduced by the doping of catalysts; for such purposes, the TiO_2_ is an excellent support for metal ions, since the nanometric particles forming it have a high surface specified in the material, where it is feasible to deposit silver [4].

The potential applications of the use of titanium dioxide in the removal of pollutants has been investigated in the photocatalytic degradation of endocrine disrupting compounds (EDCs) and is a promising approach to remove a variety of EDCs from contaminated water. Photo-catalysis mediated by metal oxide nanomaterials, such as TiO_2_, ZnO, WO_3_, ZnS, SnO_2_ and Fe_2_O_3_ and Bi_2_WO_6_, were useful to degrade endocrine disrupting contaminants [5,6,7,8,9]. Desirable attributes such as high efficiency, chemical and photo stability, low cost, commercial availability and biocompatibility of TiO_2_ makes it a most preferred and actively studied photo-catalyst to eliminate EDCs, likewise photocatalytic treatments are efficient in the removal of pharmaceuticals [10]. Some drugs such as diclofenac, propranolol, carbamazepine, and ibuprofen were treated by photolysis and photo-catalysis using immobilized TiO_2_ under simulated solar irradiation; with the processes in the absence of catalyst being insignificant compared to accelerated catalysis with light. In addition, the data experiments were treated using the LH-HW (Langmuir/Hinshelwood-Hougen/Watson) equation.

This work shows water treatment with photocatalytic processes and their coupling to the Photocatalytic Rotating Reactor (RFR). This type of reactor manages to reduce some of the main challenges of the photo-catalysis such as the use of the catalyst in suspension. Its design is part of the biological contactors and can be used as a hybrid reactor so it can be considered a viable option to treat small volumes of industrial water.

### Importance of the Investigation

There are important factors to consider when designing a photocatalytic reactor. The need to use a solid catalyst complicates the process by adding another phase to the system. In this type of reactor, it becomes evident that, besides making the contact between the reagents and the catalyst (high catalyst surface area per unit volume of the reactor) efficient, a high exposure of the catalyst to the radiation (optimal distribution of light inside the equipment) is required. A photo-reactor of rotating disks, considered a novel photo-reactive system and suitable for large scale applications, is a good example of a system that uses the supported photo-catalyst and can operate with sunlight or UV light in a continuous system.

One of the first works [11] using photocatalytic rotating reactors showed that the initial increase in reaction velocity with respect to the angular velocity of the disk is attributed to the time available per rotation. This work focuses on the development, characterization and evaluation of the TiO_2_ Rotating Disk Photocatalytic Reactor (RDPR) for the treatment of organic pollutants in water. A commercial TiO_2_-based catalyst in the form of composite ceramic balls was used as the immobilized photocatalyst on the rotating disk. The rate of decomposition of 4-chlorobenzoic acid showed Langmuir–Hinshelwood kinetics. The results obtained suggest the absence of significant mass transfer limitations at angular velocities greater than 6 rpm.

There are few current references and developments for photocatalytic rotating reactors. Recent developments [12] combined a system of titanium oxide nanotubes in a rotary disk reactor (RDPR) performing degradation tests with rhodamine B. The conversion rates were greater than 90% after a three-hour reaction. While these works only show the coupling of different forms and modification of the catalysts in a photo-catalytic system, the contributions made by these have led to improvement and a better understanding of the photocatalytic phenomena.

In the immobilization of the catalyst in a photo-catalytic rotary disks reactor, the (MLRDR) was developed [13]. The catalyst was immobilized on the disks by multilayers, and the main contributions of this work are focused on the influence of the number of layers and the volumetric flow velocity; the authors concluded that the efficiency of the reactor relies completely on these parameters.

On the other hand, the combination of photocatalytic and electrochemical treatments have generated studies based [14] on a well-developed photocatalytic fuel cell equipped with dual rotating disks for wastewater treatment. The innovation in this new device was the use of a hemoglobin on graphite cathode for in situ hydrogen peroxide (H_2_O_2_) generation. This design uses the invalid excited electron from the photo-anode and enhances the overall performance of “Rhodamine B” degradation compared with the cells using the cathode without hemoglobin. Compared to traditional photocatalytic reactors, this photocatalytic fuel cell shows greater better utilization efficiency of incident light and a higher degradation performance of organic pollutants.

Recently, innovative proposals have been developed for the removal of contaminants from the industry [15] as the system of rotary disks for post-treatment of water in the textile industry. The main contributions of this work is in the structure of the disks since they exhibit a high surface and an efficient use of UV light. The operating conditions of the disks were 20 rpm and the initial capacity of the reactor was 120 mL. In the same way [16], the development of the PRD reactor (Photocatalytic rotating disks) showed that H_2_O_2_ increases the mineralization of the orange methyl dye. The main contribution of this design is an improved capacity of treatment of 5 L, and the PRD reactor can be up–scaled for application in industrial wasterwater treatment, with a capability of advanced treatment of biodegraded wasterwater at a high efficiency.

In the removal of endocrine disrupting compounds (EDCs) such as bisphenol A, 17-ethynyl estradiol and 17-estradiol [17], there were removal efficiencies of mixed EDCs in two different scales of rotating and flat-type TiO_2_ photocatalytic reactors, and the reactor performances on removal efficiency were compared. Several operational parameters such as hydraulic retention time (HRT), initial concentrations, single and mixed compounds, UV intensities, dissolved oxygen, effect of the average solar UV intensities, and pH on EDC-removal process were demonstrated under outdoor solar irradiation. The results revealed that, for both photocatalytic reactors (rotating and flat-type), decrease in HRT increased degradation efficiency because of increased mass transfer, and the degradation efficiencies of the mixed EDCs were significantly influenced by the change of the hydraulic retention time (HRT).

## 2. Materials and Methods

### 2.1. Characteristics of RFR

**Figure 1:**
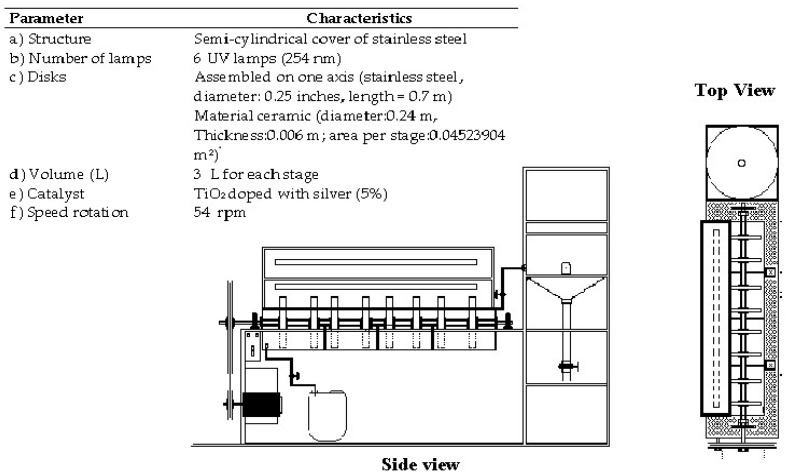
Schematic of the operation of the RFR adapted with UV lamps of λ = 254 nm, rotation speed = 54 rpm; the RFR has eight disks impregnated with TiO_2_-Ag catalyst.

The configuration and dimension of RFRs provides a high area of contact between the solid and aqueous phase; in this system, the catalyst is supported on ceramic disks, the contact between disks and aqueous phase form thin films of water on the disks; one phase is in contact with water and the other with air; the water adhering to the disk comes into contact with both the oxygen in the atmosphere and with the ultraviolet light or natural light that is irradiated by the lamps. In this design, 40% of the disks area is submerged. The main characteristics of the Rotary Photocatalytic Reactors (RFR) are described in Figure 1. The RFR is provided with a storage tank with a capacity of 50 L, which is periodically supplied to the reaction system and has control of the speed of rotation. Its operation allows for maintaining conditions continuously, and its design allows for taking samples of the progress of the reaction in each of the stages without stopping the operation of the system.

### 2.2. Synthesis and Coupling of the Catalyst TiO_2_/Ag to the RFR

Advanced oxidation process involves production of hydroxyl radical for industrial wastewater treatment. This method is based on the irradiation of UV light to photo-catalysts such as TiO_2_ for photo-degradation of pollutants. UV light is used for irradiation in photocatalytic process because TiO_2_ has a high band gap energy, which is around 3.2 eV. There can be shift adsorption to visible light by reducing the band gap energy to below 3.2 eV. Doped catalyst is one of the means to reduce band gap energy. Different methods are used for doped catalyst, which uses transition metals and titanium dioxide. Some of the transition metals change the energy level to below 3.2 eV and the adsorption shifts to visible light for degradation of industrial pollutants after being doped with titanium dioxide [18]. In this work, the silver metal is deposited in the catalyst by a process of photo-deposition; UV light has its action in the process since it reduces the metal before the calcination process in the presence of oxygen, which makes the reduction of the metal as Ag^0^ or the photo-deposition process more efficient. AgNO_3_ precursor salts were also used. The mass used was estimated based on a weight/weight ratio. Initially, the solution containing the catalyst remained for one hour in the dark phase. Then, it irradiated by four 365 nm lamps for 2 h with the addition of nitrogen (80 cm^3^/min). Water is removed by vacuum filtration, and the material is dried at a temperature of 100 °C and calcined at 550 °C. The particle size was homogenized and impregnated in wet ceramic disks with a ratio of 2 g/M^2^. The area covered by the discs was 3.5185 M^2^ representing a contact area of 387 M^2^/g available for the reaction. Finally, the impregnated disks were calcined at a temperature of 550 °C. In the same way, the calcination is a thermal process in the presence of oxygen or air that is commonly applied in the formation of photo-catalysts in order to facilitate phase transition, thermal decomposition, or the removal of a volatile fraction. Accordingly, applying calcination to doped-TiO_2_ formation can also improve its photocatalytic activity, morphology, surface area and crystallinity properties as well as the photo-catalyst’s optical absorption [19,20].

The photocatalytic activity of photocatalysts has been found to be directly and extremely proportional to dopant concentration [21]. However, beyond an optimal amount of dopant in the photocatalyst structure, the photocatalytic performance decreases [22].

### 2.3. Characterization of the Catalyst

The surface morphological analysis by secondary electrons and chemical analysis by energy dispersive spectroscopy (EDS) was performed in a Dual Beam Scanning Electron Microscope (FIB/SEM) FEI-Helios Nanolab 600 from the National Laboratory of Nanosciences and Nanotechnology Research (LINAN, San Luis Potosí, México). XRD analyses were performed with a Rigaku, SMART LAB model (LINAN, San Luis Potosí, México) using a copper tube as the X-ray source. The estimation of the band gap value (*E_g_*) was performed by UV spectroscopy using a Shimadzu UV-2450 equipment (Autonomous University of San Luis Potosi, México), provided with the ISR-2200 Integrating Sphere Attachment.

### 2.4. Photoactivity Tests

The photoactivity tests were performed with solutions of acetaminophen at different concentrations. For analysis of reaction kinetics, samples were taken at different time intervals, which were filtered with a Millipore GV membrane (pore diameter 0.22 µm, Merck, Millipore, Burlington, MA, USA). The final solution was analyzed using High Performance Liquid Chromatography (HPLC) on an Agilent 1100 Series equipment (Universidad Autónoma del Carmen, Campeche, México) with the following specifications: Column Zorbax ODS 4.6 × 150 mm, 5 μm; flow conditions: 1 mL/min, Detector: UV-Vis at 242 nm; Mobile phase: water–methanol (50/50).

## 3. Results

### 3.1. Characterization of the Catalyst (XRD, EDS and Diffuse Reflectance)

**Figure 2:**
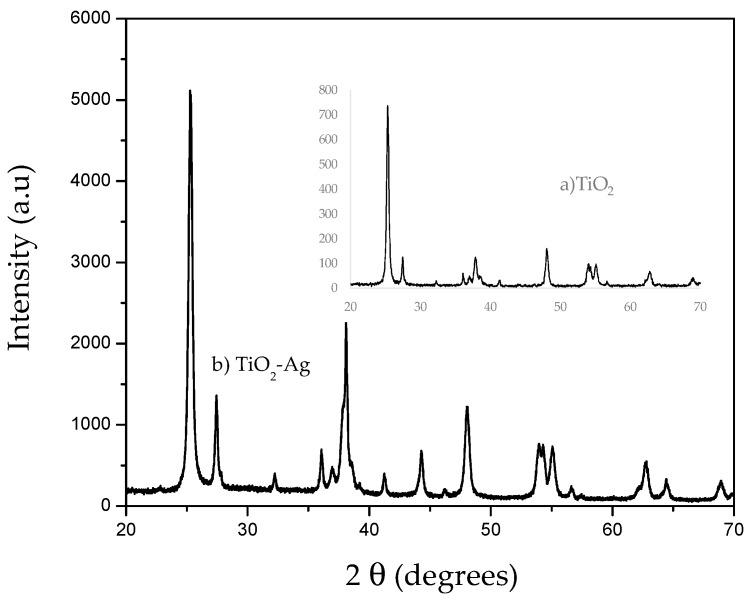
X-ray diffraction pattern of the silver nanoparticle-modified catalyst (**b**) compared to the commercial catalyst (**a**); different intensities are shown for the commercial catalysts that are attributed to the metallic silver.

The diffractogram of the catalyst is shown in Figure 2. The presence of anatase and rutile phases can be observed in the materials after silver deposition. This indicates that no structural effect on the substrate was created by the different treatments performed during the silver deposition. Differences are observed with respect to the titanium oxide without doping (Degussa P-25), with intensities at 2θ = 45, 65 corresponding to metallic silver. There is no significant variation of the percentages of anatase with respect to P-25. There are evident peaks that show the presence of silver indicating the distribution of the particles in the catalyst. The results are consistent with other studies where [23,24] defined peaks for Ag/TiO_2_ have been determined in 38.2, 44.4 and 64.5. The effect of silver species (ionic/reduced) and its concentration on the structural, textural, and catalytic properties of Ag–TiO_2_ are very significant.

**Figure 3:**
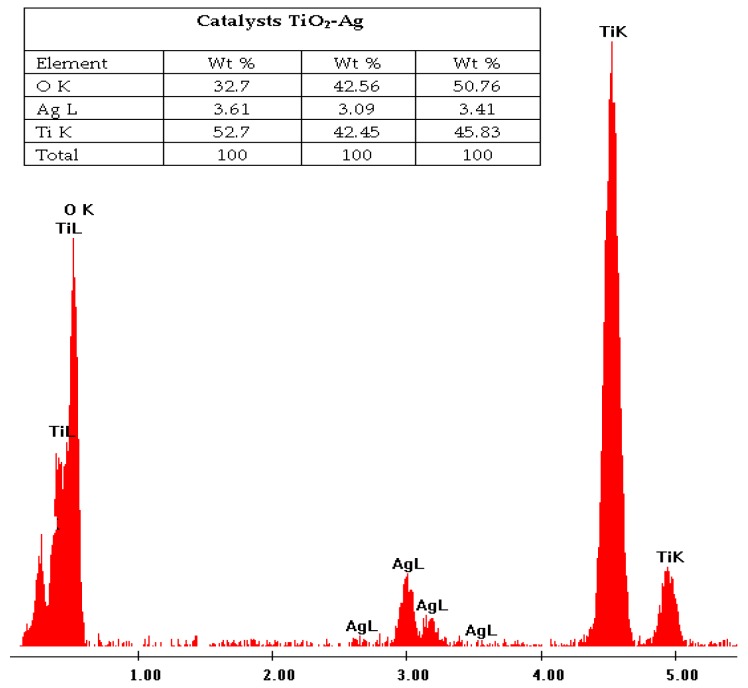
Elemental analysis of catalyst showing fractions of titanium, oxygen and silver metal. The results of three measurements made on the catalyst are shown. The average weight of the metallic silver deposited is 3.3%; the theoretical percentage was 5%.

Elemental analysis of the catalyst (Figure 3) was performed using energy dispersion spectroscopy (EDS).

**Figure 4:**
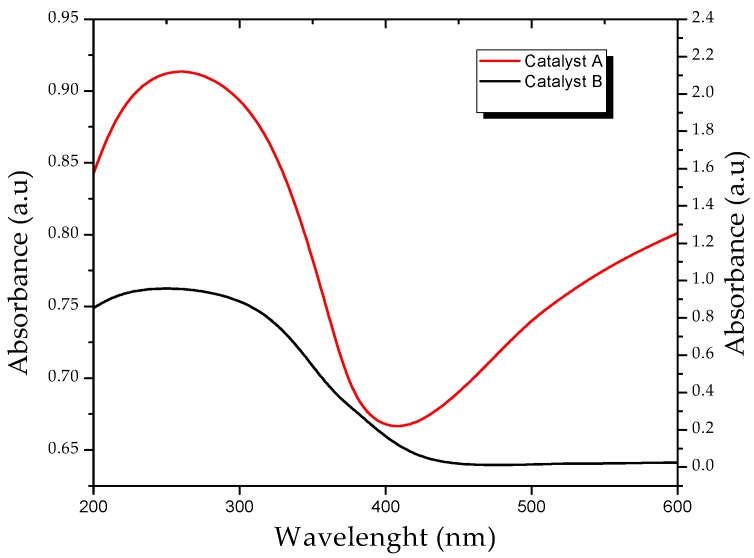
Comparison of the diffuse reflactance spectrum of the commercial catalyst TiO_2_ (**B**) with the catalyst modified with nano-silver particles (**A**).

Figure 4 shows the changes in the properties of the titanium surface by the presence of silver nanoparticles. This can improve the photocatalytic activity of the material in the visible region, In addition, a broad peak with absorption edge at 500–600 nm can also be seen in the spectrum, which is ascribed to the formation of impurity energy levels within the band gap. The addition of silver ions produces significant changes in the absorption spectrum of TiO_2_, resulting in absorbance above 400 nm showing the possibility of lower energy transitions. The valence band electrons of TiO_2_ are excited to localized energy levels created by doped silver in the band gap of TiO_2_ at longer wavelength. UV absorption spectra of Ag-doped TiO_2_ showed that an absorption peak at around 500 nm is most likely a plasmonic resonance peak related to Ag [25].

For the calculation of the band gap energy [26], the value of the intersection of the tangent line to the curve with the axis of the wavelength is taken into account. This value is used in the Planck equation that relates the energy of a photon and the frequency, taking into account the relationship between frequency and speed of light. We have Equation (8), where: *λ_g_* = Wavelength (nm), *h* = Planck constant, and *c* = Speed of light in vacuum. ${\left(Abs*hv\right)}^{\frac{1}{2}}$ is plotted as a function of hv and a line is extrapolated to the *x*-axis, which generates the estimated value of the band gap of 2.9 ev for the Ag-doped TiO_2_ compared to the commercial catalyst that is 3.1 ev:

(8) $$v=\frac{c}{\lambda_{g}},$$

(9) $$E_{g}=\frac{hc}{\lambda_{g}}=\frac{h(v\lambda_{g})}{\lambda_{g}}=hv.$$

### 3.2. Kinetics of the Reaction

The optimum rotation speed was 54 rpm. The conversion factor of acetaminophen (30 mg/L) to these conditions was 73% achieved by raising the contact between the aqueous and solid phases. The efficiency of the system was determined by the acetaminophen photo-activity tests at concentrations of 20–160 mg/L with the purpose of knowing the effect of the concentration of the substrate on the rate of degradation. Thus, for all tests, samples were taken to determine the degree of progress of the reaction. The samples were filtered and analyzed by Agilent 1100 Series High Performance Liquid Chromatography (HPLC) Column: Zorbax ODS 4.6 × 150 mm, 5 μm, flow conditions: 1 mL/min Detector: UV-Vis at 242 nm, Mobile phase: 50/50 water–methanol.

The percentage of removal was calculated with the relation:

(10) $$\%DEG=\left(1-\frac{C_{TF}}{C_{0}}\right)\times 100,$$

where *C_TF_* = is the final concentration determined by liquid chromatography and *C*_0_ = initial concentration.

**Figure 5:**
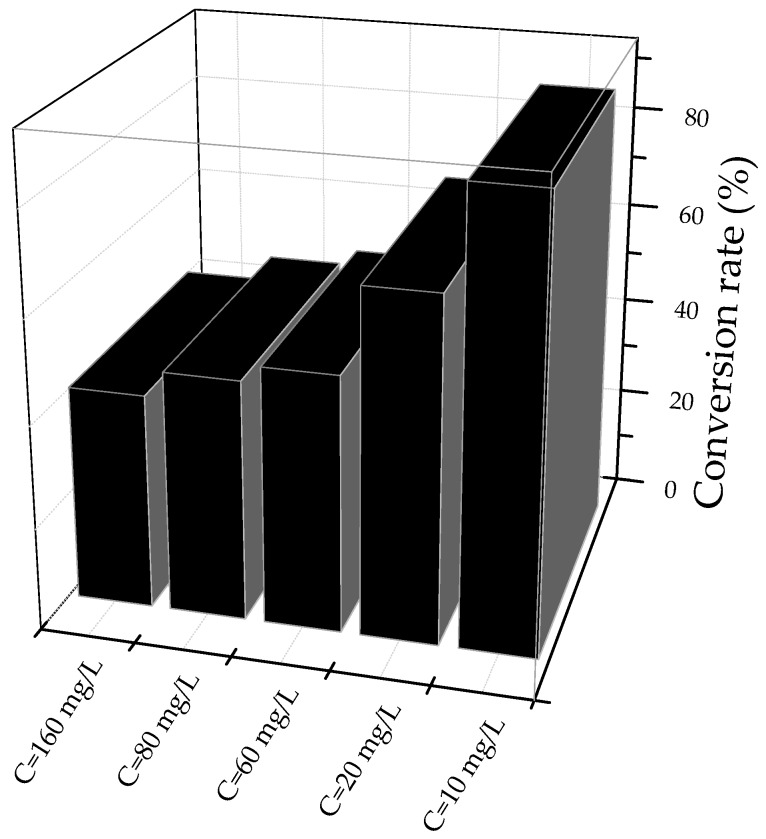
Percentages of conversion of the degradation of acetaminophen to different concentrations (catalyst mass = 1 gr/L, rotational speed = 54 rpm, radiation of λ = 254 nm, number of lamps = 6, reaction volume = 12 L, maximum reaction time = 50 h).

Figure 5 shows the conversion rates obtained by varying the reactant concentration and rotational speed of the disks.

**Figure 6:**
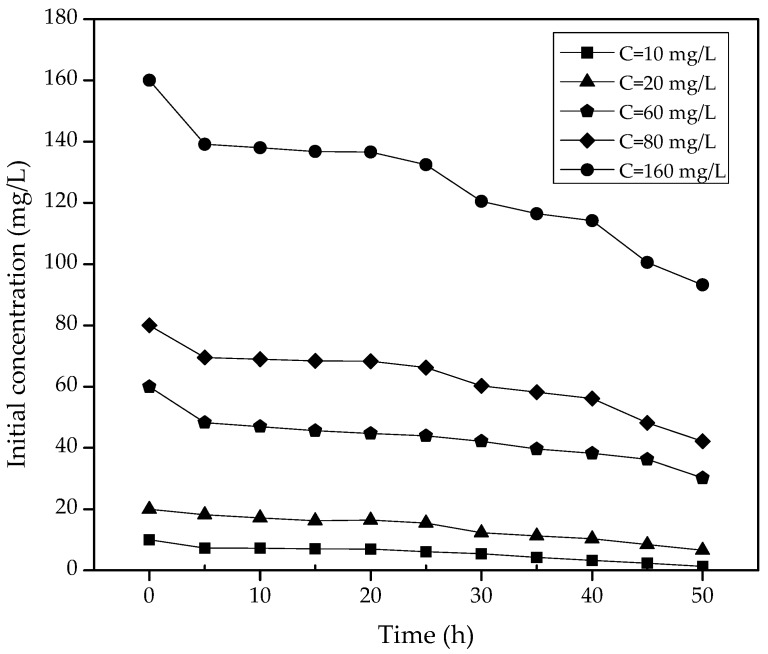
Profile of the degradation of acetaminophen followed by HPLC (catalyst mass = 1gr/L, rotational speed = 54 rpm, radiation of λ = 254 nm, number of lamps = 6, reaction volume = 12 L, maximum reaction time = 50 h).

**Figure 7:**
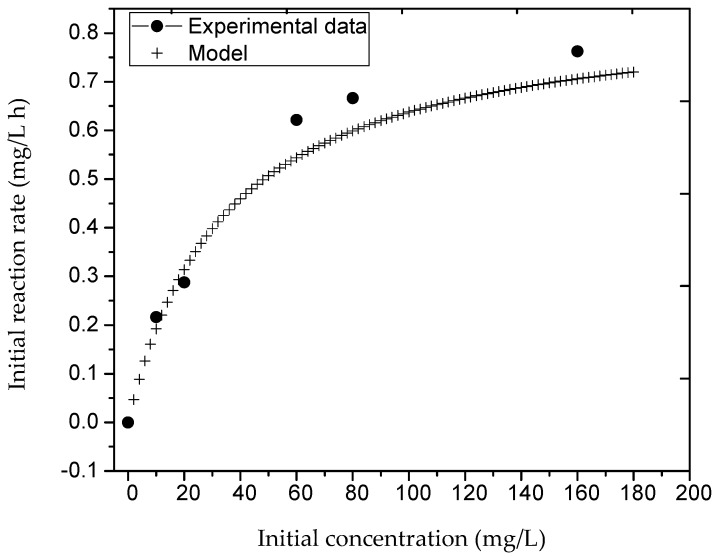
Heterogeneous LH catalysis model applied to the degradation of acetaminophen in an RFR reactor. The solid line represents the model and the points represent experimental data (catalyst mass = 1 gr/L, rotational speed = 54 rpm, radiation of λ = 254 nm, number of lamps = 6, reaction volume= 12 L, maximum reaction time = 50 h).

The initial concentration is of paramount importance in the photocatalytic reactions since there is a high dependence on the concentration on reactions analyzed under the behavior of the first order kinetics. Figure 6 shows the profile of the acetaminophen concentration at different reaction times obtained by liquid chromatography. Photocatalytic oxidation kinetics of organic compounds can be successfully modeled using the LH-HW equation to describe a correlation between degradation rate constants and initial concentration [27]:

(11) $$-r_{AC}=-\frac{dC_{AC}}{dt}=\frac{K_{1}C_{AC}}{1+K_{2}C_{AC}},$$

(12) $$-r_{AC}=\frac{0.024715C_{AC}}{1+0.02878C_{AC}},$$

where *K*_1_ is the pseudo-first order rate constant (min^−1^) and *K*_2_ is the adsortion constant of acetaminophen over TiO_2_ surface in aqueous enviroments. The values of the kinetic constants *K*_1_ = 0.0247157 (min)^−1^ and *K*_2_ = 0.02878 (mg/L)^−1^ were obtained by nonlinear regression using the Levenberg–Marquardt Algorithm. The experimental data was fitted to the LH-HW catalytic model as shown in Figure 7.

Acetaminophen has been one of the most studied pharmaceuticals under advanced oxidation processes recently [28]. Hollow core-shell mesoporous TiO_2_ microspheres were synthesized by a template-free solvothermal route for efficient photocatalytic degradation of acetaminophen data revealed a micrometer-sized mesoporous anatase TiO_2_ hollow sphere with large surface area and efficient light harvesting. The conversion fraction of the pharmaceutical increased from 88% over commercial Degussa P25 TiO_2_ to 94%. The use of doped materials has also been reported [27]. This potassium peroxodisulfate K_2_S_2_O_8_ can act as a dopant for the titanium oxide synthesized by sol gel, and it has been reported that there is a 100% conversion of acetaminophen under these conditions.

In the same sense, our research group has reported conversions greater than 90% of acetaminophen [29] with un-doped TiO_2_. The difference between our current study and the previous ones is the scale of work and the ability to treat a larger volume of water in a semi-continuous way with the RFR.

**Figure 8:**
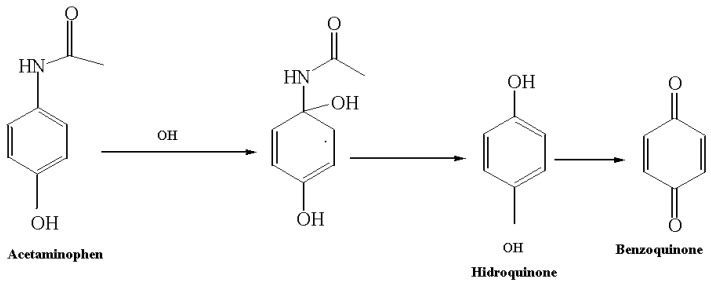
Major organic products (OIP) determined by co-injection of standards with liquid chromatography on the degradation of acetaminophen in an RFR reactor with titanium-silver catalysts.

They have been identified as intermediate products of the photocatalytic reaction of acetaminophen to nitrophenol, aminophenol, hydroquinone and numerous carboxylic acids by analytical techniques such as infrared spectroscopy (FT-IR) and high-resolution liquid chromatography (HPLC) [30]. In this work, under the technique of co-injection of standards in the conditions in which the separation of acetaminophen by liquid chromatography was carried out, two additional peaks to acetaminophen were identified that correspond to the structure of the hydroquinone with a retention time of 3.51 min and benzoquinone with a retention time of 3.78 minutes. With the available tools, it was possible to verify that the degradation path follows a hydroxylation pathway, for which a displacement of the functional group of acetamide by an HO• adical initially occurs (Figure 8).

## 4. Conclusions

In summary, a type of RFR reactor was developed, which is a viable option for the treatment of water with organic pollutants on a semi-industrial scale. The additional advantages conferred by the silver-doped catalyst could be applied in the inactivation of bacteria that can be coupled to a biological post-treatment. Furthermore, the results of the application of the photocatalytic treatment with the treated volumes could be increased with the use of hydrogen peroxide or another oxidant to accelerate the degradation of the organic matter and reduce the hydraulic residence times, the proposal is presented in a reactor with a capacity to treat up to a volume of 50 L continuously, unlike conventional photocatalytic reactors on a laboratory scale that usually treat very small volumes.
